# Isolation, Characterization and Structural Elucidation of Polybutylene Terephthalate Cyclic Oligomers and Purity Assessment Using a ^1^H qNMR Method

**DOI:** 10.3390/polym11030464

**Published:** 2019-03-11

**Authors:** Emmanouil D. Tsochatzis, Joao Alberto Lopes, Margaret V. Holland, Fabiano Reniero, Hendrik Emons, Claude Guillou

**Affiliations:** European Commission, Joint Research Centre (JRC), Via Enrico Fermi 2749, I-20127 Ispra (VA), Italy; Emmanouil.TSOCHATZIS@ec.europa.eu (E.D.T.); Margaret.HOLLAND@ec.europa.eu (M.V.H.); Fabiano.RENIERO@ec.europa.eu (F.R.); Hendrik.EMONS@ec.europa.eu (H.E.); Claude.GUILLOU@ec.europa.eu (C.G.)

**Keywords:** cyclic oligomers of polybutylene terephthalate (PBT), preparative HPLC (p-HPLC), High Resolution Mass Spectrometry (HR-MS), Nuclear Magnetic Resonance (NMR), qNMR purity assessment

## Abstract

The use of polybutylene terephthalate (PBT) as a food contact material is increasing over the last years. Typical contaminations in the final PBT product include its cyclic oligomers, which are allowed as additives in food contact plastics according to Regulation (EU) No. 10/2011. Their investigation is currently limited by the lack of analytical standards and physical-chemical information. Therefore, four PBT cyclic oligomers have been isolated and purified from a PBT raw material with an automated preparative HPLC-DAD system. Comprehensive characterization of the compounds was performed using Ultra-High Performance Liquid Chromatography (UHPLC) with high resolution time-of-flight mass spectrometry, Fourier-Transform Infrared spectroscopy (FTIR), Differential Scanning Calorimetry (DSC) and Nuclear Magnetic Resonance (NMR) spectroscopy. The purity of each oligomer was assessed using a ^1^H qNMR method and ranged from 96.1% to 97.0% for PBT tetramer and trimer respectively. The availability of pure and well characterized PBT cyclic oligomer standards will facilitate future studies of release from plastic food packaging materials.

## 1. Introduction

Polyesters play an important role in the production of food contact materials (FCMs). In addition to polyethylene terephthalate (PET)—a plastic material that has been used for many years in food packaging—different types of polyesters are being introduced at an increasing rate in a number of applications. A relatively novel material in the area of FCMs is polybutylene terephthalate (PBT), which is currently used in kitchenware utensils, microwaveable dishware, coffee capsules and beverage cups [1]. This polymer is usually obtained by step-growth polymerization methods and nuclear magnetic resonance (NMR) is considered the most powerful technique for its structural characterization [2].

A high melting point and viscosity are among the most important physical properties of thermoplastics like polyesters. However this may also cause undesirable characteristics, like a strong tendency to crystallize that consequently leads to a loss of transparency in the final product [2]. One way to overcome this problem would be to develop thermoplastic resins that can be polymerized reactively, like thermosetting resins, while at the same time behave like thermoplastics. Another option would be to add cyclic oligomers, like those from PBT, during the production of the polymer [1,2,3].

In Europe a mixture of PBT cyclic oligomers is already allowed as an additive to food packaging materials according to the EU Regulation No. 10/2011. According to Table I in the Annex 1 of the aforementioned EU Regulation, FCM No. 885 is a mixture composed of the dimer (M = 440 Da, 33%), the trimer (M = 660 Da, 39%), the tetramer (M = 880 Da, 12%) and the pentamer (M = 1100 Da, 13%). It can be used as an additive in some FCM polymers (PET, PBT, polycarbonate (PC), polystyrene (PS) and rigid polyvinyl chloride (PVC)) but can only be used at mass fractions up to 1% *w*/*w* [4,5]. Additionally, the restriction requires that these plastics can only be used for contact with aqueous, acidic and alcoholic foods and for long-term storage at room temperature [4,5]. 85% of the FCM No. 885 mixture has a molecular weight below 1000 Da and is considered to be highly lipophilic nature with a log P_ow_ of 3.5 [4].

For the development and validation of an analytical method the availability of standards for each of the target analytes is essential. When no standards of known purity are commercially available, the two most commonly used approaches are either to synthetize or isolate them from a known matrix. Some examples exist already in the literature related with FCM oligomers [6,7,8]. However, to the best of the author’s knowledge, no analytical standards for the individual PBT cyclic oligomers are yet commercially available. Therefore, PBT cyclic oligomers from dimer to pentamer were isolated in this study from a premixed raw polymeric material, after optimizing both dissolution and separation conditions, by applying preparative HPLC (p-HPLC). Structural elucidation for the four oligomers was carried out by 1D and 2D ^1^H and ^13^C-NMR, UHPLC-qTOF-MS and FTIR spectroscopy. Additionally, DSC analysis and solubility studies were performed. The purity of each oligomer has been assessed by using a quantitative ^1^H NMR (q-NMR) method using a certified reference material as internal standard. This paper presents a comprehensive study of these specific oligomers.

## 2. Materials and Methods

### 2.1. Chemicals

Solvents such as ethanol (EtOH), methanol (MeOH), 2-propanol (IsOH), acetone (Acet), acetonitrile (MeCN), dimethylformamide (DMF), dimethylacetamide (DMA), dimethyl sulfoxide (DMSO), tetrahydrofuran (THF), dichloromethane (DCM), chloroform (CHCl_3_), 2,2,2-trifuoroethanol (TFE) and 1,1,1,3,3,3-hexafluoro-2-propanol (HFIP) were all of CHROMASOLV grade and supplied by Sigma Aldrich (Steinheim, Germany). Formic acid was LC-MS grade and ultrapure water (18.2 MΩ) was used in the preparation of solutions. Chloroform-d (99.8%) was provided by Aldrich, while the certified qNMR standard Dimethyl sulfone-DMSO_2_ (TraceCERT) was supplied by Sigma Aldrich (Steinheim, Germany). The raw polymeric PBT mixture was supplied by the producer of FCM No. 885 (PolyOne France S.A.S, Paris, France), available at the European’s Union Reference Laboratory for Food Contact Materials (EURL-FCM) database for additives. Titan3 17 mm PTFE, 0.2 μm membrane filters were supplied by CPS Analitica (Milan, Italy).

### 2.2. Sample Pre-Treatment

For the sample preparation a screening process was performed to evaluate the solubility of the starting material in different organic solvents in order to obtain the maximum possible amount of the oligomer in solution. After completing these tests (Section 3.1) HFIP was selected as the solvent with the best solubilizing properties.

For the preparation of the working solution, a portion of 1 g of material was dissolved in 5 mL of HFIP and placed in a sonicator for 20 min at 30 °C. After complete dissolution, working solutions were prepared by performing a 100 fold dilution of the sample with acetonitrile. 

### 2.3. HPLC-DAD Analysis

An Agilent Technologies 1200 series HPLC system (Waldbronn, Germany), equipped with a thermostatic column compartment, an auto-sampler and a diode array detector (DAD) was used. The chromatographic column was a Thermo Scientific HyPURITY C_18_ 150 × 3.0 mm, 5 μm particle size (Thermo Fisher Scientific Inc., Columbus, OH, USA) which was thermostatically stabilized at 40 °C. Separation of the target analytes was performed with a linear gradient elution program, using a mobile phase of a mixture of acetonitrile (A) and water (B). The gradient elution program was optimized as follows: 60% B t = 0 min, to t = 20 min 20% B, followed by a gradient decrease of B to 60% (t = 21 min) and an equilibration post-time of 4 min to reach the initial conditions prior to the next injection. The injection volume was 10 μL, the flow 1 mL/min and the ultraviolet (UV) spectra were recorded from 200 to 400 nm). The 240 nm channel was selected for the analysis as it represents the maximum absorbance wavelength for the PBT oligomers.

### 2.4. UHPLC–qTOF-MS Analysis

A UHPLC system (Agilent 1290) was interfaced with a quadrupole Time-Of-Flight (TOF) mass spectrometer detector (Agilent 6540 UHD Accurate-Mass, Agilent, Waldbronn, Germany), using an ESI interface, operating in both positive and negative ionization modes. The source operated at 325 °C and nitrogen was used as the drying gas, at 2.8 bar, as well as nebulizing gas at a flow of 10 L min^−1^. The injection volume was 5 μL and the TOF-MS detector was set to acquire MS data over an *m*/*z* range of 100 to 1600. A capillary voltage of 4 kV was used in positive and 3 kV in negative ESI mode.

The separation was performed in a Waters analytical column BEH C_18_ 100 × 2.1 mm, 1.7 μm particle size (Waters, Milford, MA, USA) at a flow rate of 200 μL min^−1^. The column was thermostatically controlled at 40 °C and the mobile phase consisted of water with 0.1% formic acid (A) and methanol with 0.1% formic acid (B). A gradient program was applied, starting from 50% B changed linearly to 95% B at 25 min followed by an isocratic elution for 4 min (t = 29 min). An equilibration of 1 min was set for the mobile phase to reach initial conditions.

An MS/MS fragmentation experiment was performed based on the precursor ion (*m*/*z*) of each of the isolated cyclic PBT oligomers. The optimum collision energy (eV) for each of the target analytes was optimized, performing experiments ranging from 0 to 100 eV.

### 2.5. p-HPLC Isolation of PBT Oligomers

An Agilent 1290 series preparative system, equipped with a DAD (Agilent Technologies, Palo Alto, CA, USA), an automated fraction collector and a Zorbax Eclipse XDB C18 PrepHT column (21.2 mm × 150 mm, 5 μm, Agilent Technologies, Waldbronn, Germany) was used. An isocratic elution was applied with an acetonitrile:water ratio of 9:1 *v*/*v* as mobile phase. The column temperature was 40 °C and the flow rate at 10 min^−1^. Injection volume was 500 to 700 μL of each working solution (Section 2.2).

The fractions containing the target compound were collected by an automatic collector based on the UV spectrum taken and the chromatogram recorded at 254 nm. The collected fractions were combined and the solvents were removed using a rotary evaporator, followed by further drying under a stream of nitrogen at 45 °C. Collected fractions were diluted and reinjected in the HPLC-DAD system in order to assess their purity.

### 2.6. NMR Analysis

Compounds were characterized by mono-dimensional ^1^H and ^13^C APT experiments, as well as bi-dimensional ^1^H/^13^C HSQC, ^1^H/^13^C HMBC and ^1^H/^1^H COSY experiments using standard Bruker experiment sequences, which were performed to confirm the molecular structure. 

Each PBT oligomer was dissolved in 600 µL of deuterated chloroform and TFA (8:1, *v*/*v*), shaken in a vortex and placed in a 5 mm NMR tube. The experiments were performed on a Bruker (Rheinstetten, Germany) Avance 600 (nominal frequency 600.13 MHz) equipped with a 5 mm cryo-probe. The spectra were recorded at 298 K using a 90° flip angle, an acquisition time of 3.0 s (64k data points) and a total recycling time of 4.0 s. A spectral width of 20 ppm with 256 scans and no sample rotation in the *DQD* acquisition mode were applied. Prior to Fourier transformation a 0.5 Hz line-broadening factor was applied and all spectra were phase- and baseline-corrected using Bruker Topspin 3.2 software.

Chemical shifts (δ) for ^1^H and ^13^C NMR spectra are reported in parts per million (ppm) relative to the internal residual solvent signal of CDCl_3_-d: 7.2 ppm for ^1^H and 77 ppm for ^13^C APT.

All samples were weighed on a Mettler Toledo (Columbus, OH, USA) digit balance of ±0.01 mg (manufacturer’s stated uncertainty). For the qNMR analysis, an accurate amount of each oligomer was weighted and mixed with an accurate amount of the internal standard (app. 3:1 *w*/*w*) in deuterated chloroform and TFA (8:1, *v*/*v*). Each oligomer was analysed 10 times, under the same NMR conditions above mentioned apart from the relaxation delay (D1), which was increased to 30 s to receive a fully relaxed NMR spectrum of the internal standard.

### 2.7. Fourier-Transform Infrared Spectroscopy (FTIR)

All spectra were acquired using the Attenuated Total Reflectance (ATR) mode with a FTIR spectrometer (spectrum 2000, Perkin Elmer, Waltham, MA, USA). Spectra were acquired in the scan range between 4000.00–530.00 cm^−1^, with a resolution of 4.00 cm^−1^ and a total of 8 scans for each sample that were averaged to eliminate background noise. Samples were analysed without any preparation and the data acquisition and processing were managed by the Spectrum™ software (Perkin Elmer, Waltham, MA, USA). The four isolated oligomers, the initial raw polymeric mixture and a PBT polymeric material were analysed.

### 2.8. Differential Scanning Calorimetry and Melting Point Assessment

A TA Instruments Model Q100 (Newcastle, DE, USA) equipped with an auto-sampler was used for DSC analyses. After sample introduction (from 3 to 5 mg in aluminium pans for each oligomer) and temperature equilibration, the sample was heated from −20 °C to 300 °C at 30 °C/min (1st cycle), cooled to −20 °C at 30 °C/min (2nd cycle) then heated up to 300 °C at 30 °C/min. The heating scans were performed under a constant gas flow of nitrogen (50 mL/min). 

The melting points of the pure substances have been assessed using a Buchi melting point instrument (Buchi Labotechnik AG, Flawii, Switzerland). A program has been set starting from a heating rate of 10 °C/min, up to temperature of 160 °C, followed by a rate of 1 °C/min.

## 3. Results and Discussion

### 3.1. Solubility Studies of Starting Material

The solubility of the raw material was investigated in order to select the most appropriate solvent to be used in the LC analysis. This study was based on the Joint Food and Agriculture Organization of the United Nations (FAO) and World Health Organization (WHO) Expert Committee of Food Additives protocols [9,10]. The same document also provides different solubility classifications for each solubility range of amount of material per volume of solvent [9,10]. 

Briefly, portions from 2.0 to 50 mg of the initial material were dissolved in different volumes of 13 solvents, namely MeOH, EtOH, IsOH, Acet, MeCN, DMF, DMA, DMSO, THF, DCM, CHCl_3_, TFE and HFIP. The raw material was completely soluble in both HFIP and CHCl_3_, while only partial solubility was achieved in acetonitrile, DCM, THF, DMA, DMF and very slight solubility in EtOH, IsOH. The material was insoluble in MeOH and Acet. The most effective solvent appeared to be HFIP and so it was selected for the dissolution of the material prior to p-HPLC.

### 3.2. Preparative HPLC

**Figure 1:**
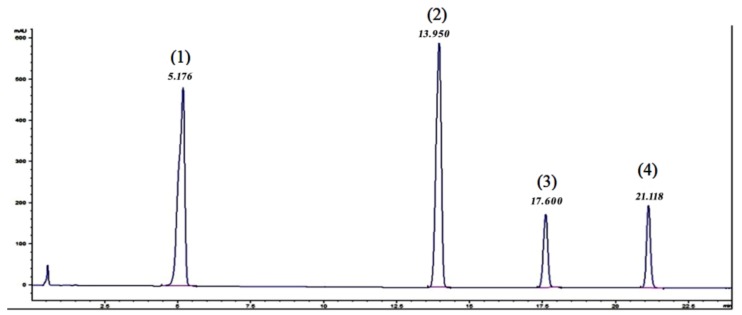
HPLC-DAD chromatogram of the raw material (Retention times: 5.18 min PBT cyclic dimer (**1**); 13.95 min PBT cyclic trimer (**2**); 17.60 min PBT cyclic tetramer (**3**); 21.12 min PBT cyclic pentamer (**4**).

In order to evaluate the existence of the target analytes, their proportion, as stated by the producer and the purity of the raw material, as well as potential matrix interferences, aliquots of 5 μL of the working solutions in HFIP were analysed with both HPLC-DAD and UHPLC-qTOF-MS. The HPLC-DAD chromatogram for the raw PBT polymeric mixture is presented in Figure 1. 

No particular interferences were visible in these chromatograms, which have been confirmed with the UHPLC-qTOF-MS. Very small traces of the PBT hexamer were identified in the mixture. These analysis also indicated the possibility of using a C_18_ semi-preparative or preparative column for the isolation of the target PBT oligomers, as the separation of the peaks was acceptable, with resolution factors (R_s_) higher than 5 in all cases.

**Figure 2:**
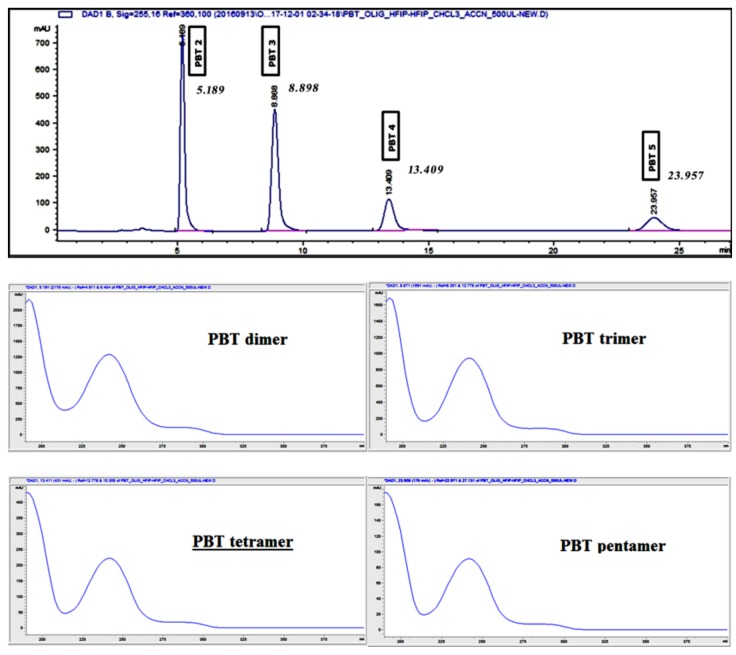
p-HPLC-DAD chromatogram of the raw polymeric material. Ultraviolet (UV) spectra for each oligomer are also presented (λ = 200–400 nm).

MeCN and MeOH in mixtures with water were tested as mobile phases. The separation of the PBT oligomers with methanolic mobile phases were poor, with very low signal for the PBT cyclic dimer and trimer and no signal at all for the tetramer and the pentamer at the concentration levels tested. This could be explained by the low miscibility of methanol with HFIP and a lower solubility of the oligomers in MeOH than in MeCN. MeCN showed better separation efficiency than methanol, because of its better miscibility with HFIP and a higher solubility of the oligomers at the studied concentration levels. Based on the above-mentioned findings, optimized for the analytical column separation, the method was scaled up for separation in the preparative column. The resulting chromatogram is presented in Figure 2. 

The p-HPLC system allowed only the use of isocratic separations. Therefore, as first the mobile phase has been adapted. A higher percentage of acetonitrile in water was selected (9:1) and the flow rate was increased 10 fold (from 1 mL min^−1^ to 10 mL min^−1^). From Figure 2 it can be seen that the chromatographic resolution was very good and on par with the obtained with the C18 analytical column, especially taking into consideration the sample volumes/concentrations being injected (500 to 700 μL of a 0.1 g mL^−1^). These results confirmed a successful scale-up of the separation.

In order to obtain considerable amounts of each oligomer a number of injections in the p-HPLC had to be performed. Each chromatographic run was divided in 4 fractions, each one corresponding to one of the isolated oligomers and they were collected using an automated fraction collector. All the collected fractions, corresponding separately to each of the isolated oligomers, were combined and evaporated to dryness, using a rotary evaporator. Purified oligomers fractions were further dried overnight in an oven at 90 °C. The isolation yield of the total amount of oligomers recovered from the initial amount of the raw material was 71% (*w*/*w*).

Solubility studies were also performed for each of the isolated oligomers, following the approach presented in Section 3.1. Results are given in Table 1. 

Some differences between the solubility of the oligomers were found. HFIP is the best solvent for all four of them, followed by TFE and CHCl_3_. As these solvents cannot be directly used in HPLC systems, dilutions of stock solutions should be prepared with HPLC compatible solvents like MeCN, always paying attention to the potential precipitation of the oligomers. 

### 3.3. UHPLC-qTOF-MS Analysis and HR-MS Spectra

**Figure 3:**
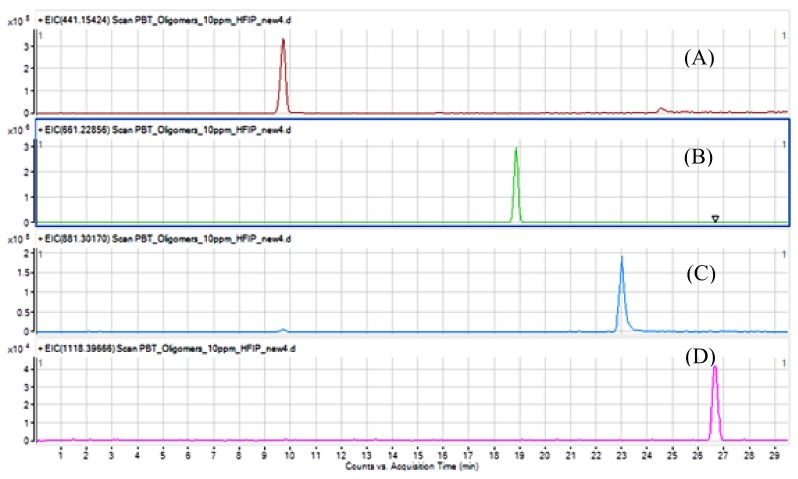
UHPLC-qTOF-MS chromatograms of 2 μg mL^−1^ cyclic PBT oligomers: (**A**) dimer; (**B**) trimer; (**C**) tetramer and (**D**) pentamer.

The target purified analytes have been measured by the UHPLC-qTOF-MS method to confirm their purity Working solutions were prepared in HFIP for each substance at a concentration of 100 μg mL^−1^. Then a dilution to 2 μg mL^−1^ was made with H_2_O:MeCN 1:1 *v*/*v* was made and 5 μL were injected in the system. The obtained chromatograms are presented in Figure 3.

The chromatographic separation of the 4 cyclic oligomers is very good. Solubility and miscibility with solvents and mobile phases must be taken into consideration, as potential precipitation in the injection port or in the column may occur. The purity of each substance was estimated by analysing the chromatograms using the peak area normalization method (oligomer peak as a percentage of the sum of the areas of all the peaks found) [11]. The relative purity for each oligomer ranged from 95% (pentamer) to 97% (dimer and timer). Typical observed impurities consist mainly of one of the other oligomers.

**Figure 4:**
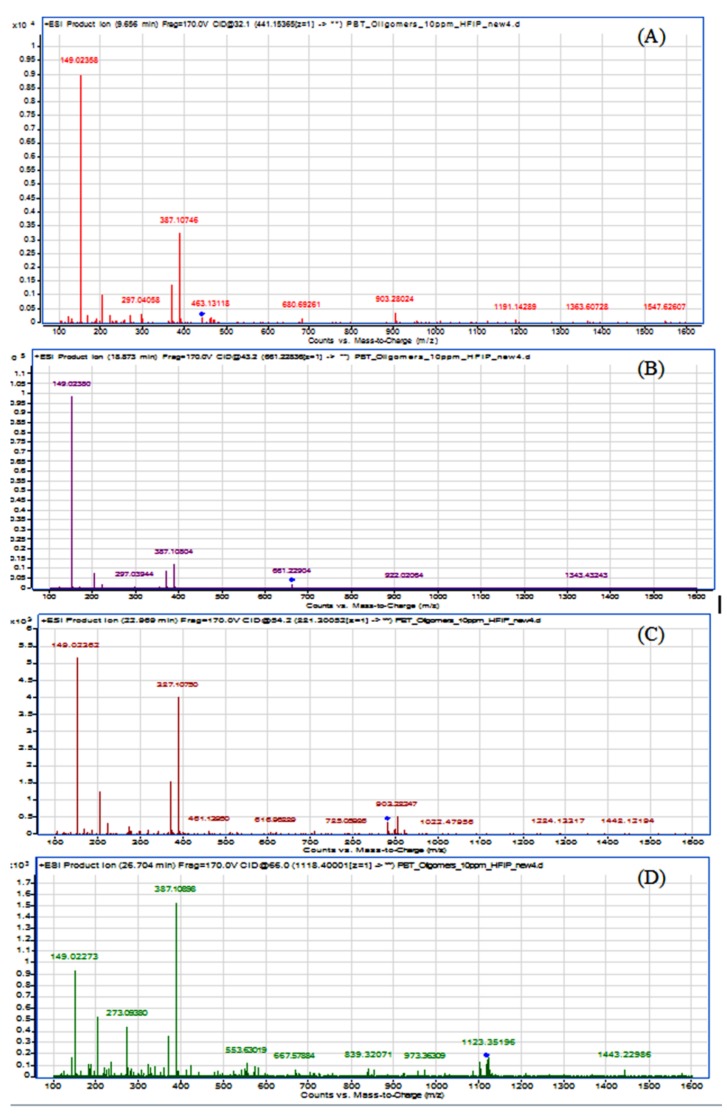
Acquired HR-MS^2^ spectra of the PBT cyclic oligomers (**A**) dimer; (**B**) trimer; (**C**) tetramer and (**D**) pentamer. Precursor ions for each oligomer have been flagged with blue dots.

Moreover, an MS^2^ experiment was performed to evaluate the oligomers fragmentation from their respective precursor ions. Results are presented in Figure 4. 

It can be observed from the deconvolution of the spectra that all the oligomers showed a similar fragmentation pattern. The precursor ions (*m*/*z*), corresponding to protonated forms (M-H^+^) for PBT cyclic dimer, trimer, tetramer and pentamer were 441.154 (463.131), 661.228, 881.301 (903.280) and 1101.400 (1123.352), respectively. In case of dimer, tetramer and pentamer, the ions (*m*/*z*) 463.131, 903.280 and 1123.352, representing the respective oligomers precursor ion plus sodium (M-Na^23+^), were more abundant than the protonated (M-H^+^) ones. It could be concluded from the fragmentation that there is a linear increase in the collision energy (eV) from the dimer to the pentamer, namely from 32.1 eV via 43.2 eV and 54.2 eV to 66.0 eV. This observation is in line with the changing chemical structure of these molecules, as with an increasing molecular mass and ring size higher collision energies are normally required for molecule fragmentation.

Additionally, the dimer fragmented to an open-dimer molecule minus a butylene group ion of 387.107 (M = 387) and in the most abundant m/z ion, the one for terephthalic acid (M = 149). The same pattern could be observed for the remaining oligomers but with the pentamer presenting instead the signal at *m*/*z* 387.109 as the most abundant. The results of the present study using ESI (+) ionization (combined with qTOF-MS) are in correspondence with a similar study regarding the MS fragmentation patterns and characterization of PBT cyclic oligomers from Bryant and Semlyen using APCI ionization [12]. It was possible to identify the *m*/*z* 149.024 signal (resulting from the terephthalic acid minus the –OH group) and *m*/*z* 387.108 (representing the hydrated dimer ring). The dimer ring fragment *m*/*z* 369 was also observed but less abundant than its hydrated form. Additionally, a fragment with *m*/*z* 203, corresponding to the butyl ester of the terephthalic acid, was also identified. Its intensity was however less abundant than that reported by Bryant and Semlyen, probably due to the different ionization mode used in both studies [12].

### 3.4. FTIR and DSC Analysis

**Figure 5:**
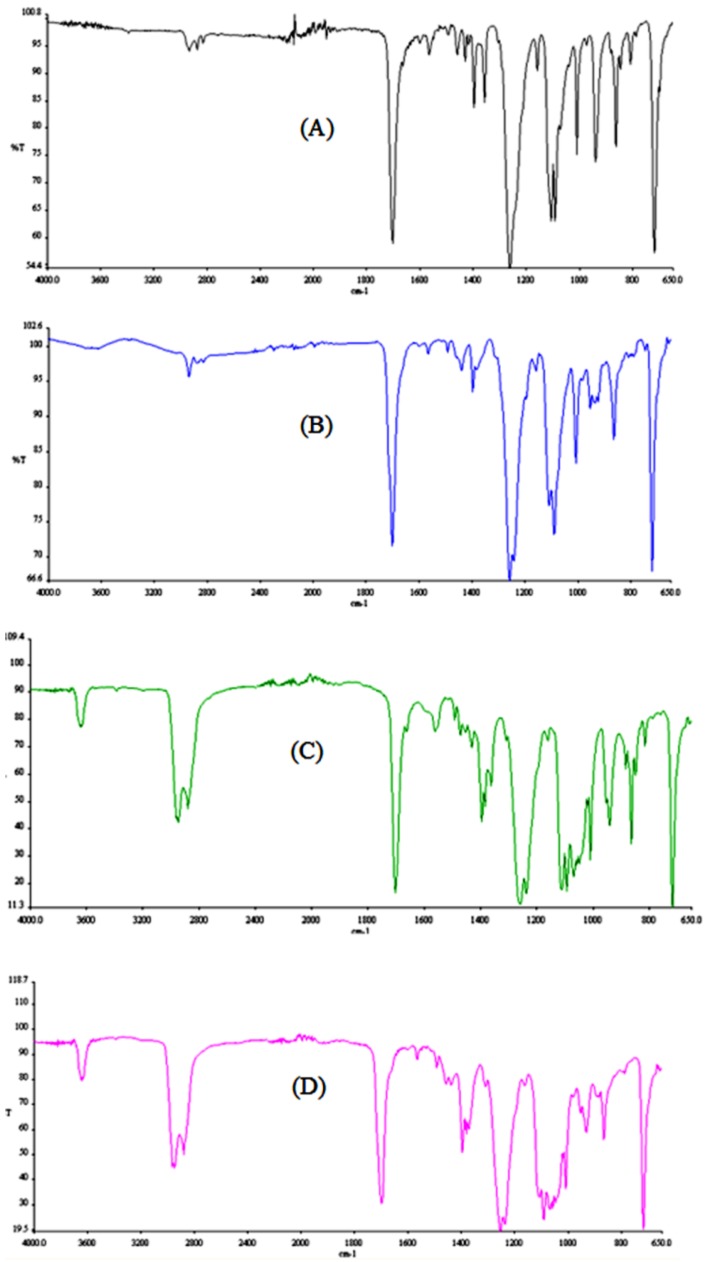
Fourier transform infrared (FTIR) spectra of the isolated PBT cyclic oligomers isolated (**A**) dimer; (**B**) trimer; (**C**) tetramer and (**D**) pentamer.

The FTIR spectra were acquired in the 4000–650 cm^−1^ range and are presented in Figure 5.

The alkyl group band at 3000 cm^−1^ can be seen in all of the oligomers’ spectra. This band increased in intensity with the increasing number of alkyl chains, from 2 chains (from 1,4-butanediol) in the dimer to 5 chains in the pentamer. It could also be noticed that this band appears as a double peak, where the second signal represents the aromatic C-H stretching (3060 cm^−1^). The increased splitting of the band is due to an increase in the number of benzene rings of the different oligomers. All the oligomers showed the typical carbonyl C=O stretching at 1720 cm^−1^. The results are similar to the FTIR results reported by Holland et al. for the respective PET cyclic oligomers [13].

For all of the oligomers the spectra show bands at approximately 1465 cm^−1^, corresponding to the bending vibrations of the methylene groups and bands at 1408 cm^−1^, corresponding to the C-C stretching vibration in the benzene rings. These are typical signals of polyester-type structures and have been identified before [14]. Again, the dimer presents much sharper bands for both wavelengths and can clearly be differentiated from the other oligomers. The tetramer and the pentamer could be distinguished from the other two oligomers (the higher number of methylene groups and benzene rings contribute to broader bands) but cannot be differentiated from each other. The C-O-C stretching, between 1000 cm^−1^ and 1150 cm^−1^, also shows the aforementioned effect.

The melting points (*T*_m_) and of the four oligomers and the PBT polymer, as determined by DSC are presented in Table 2. *T*_m_ obtained with a melting point apparatus are also shown. 

The obtained *T*_m_ values for the trimer and tetramer are in line with what has been described by Wu et al. [15]. For the dimer a difference of 2.8 °C for the *T*_m_ was observed. Wu et al. obtained the PBT oligomers via synthesis and purification but did not specify the purity. The DSC results of this work are in line with the *T*_m_ values obtained using a classical melting point apparatus. 

### 3.5. NMR Analysis

#### 3.5.1. Characterization

**Figure 6:**
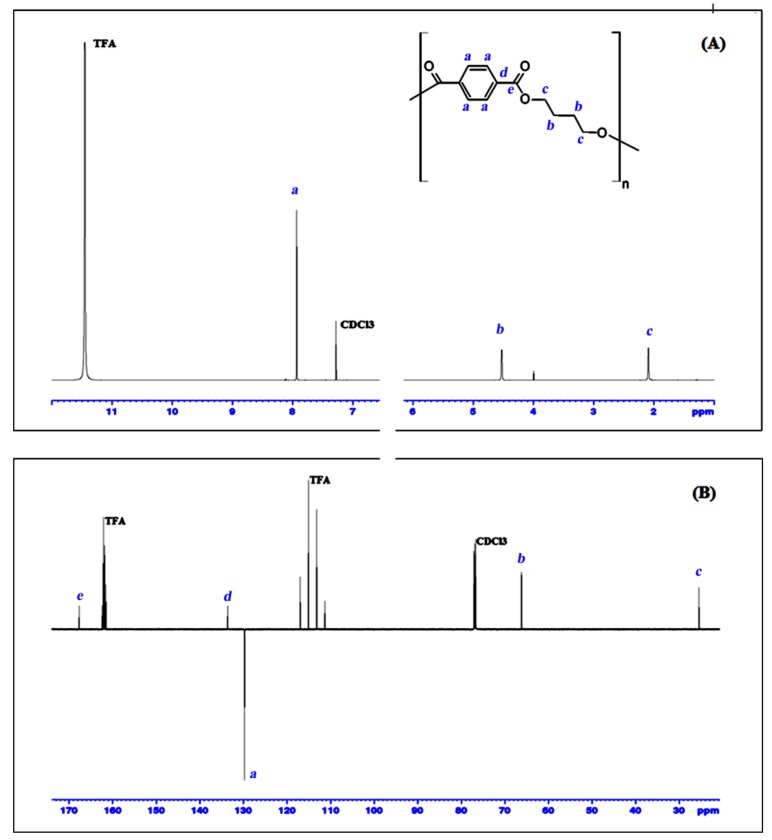
^1^H NMR spectrum (**A**) and ^13^C APT NMR spectrum (**B**) of the PBT cyclic dimer, with signals assigned to corresponding atom’s position in the molecule (a, b, c, d, e), recorded in CDCl_3_/TFA.

The chemical structure of the PBT cyclic dimer and the ^1^H NMR and ^13^C NMR spectra, along with the respective signal assignments is given in Figure 6. The chemical shifts for the 4 studied PBT cyclic oligomers are summarized in Table 3 and all the related spectra, either monodimensional ^13^C- and ^1^H NMR for the all PBT oligomers and bidimensional of the PBT dimer, are presented as supplementary information (Supplementary Information, Figures S1–S3).

From Figure 6 and Table 3 can be seen that the ^1^H NMR data are perfectly consistent with the work of Martinez de Ilarduya and Munoz-Guerra [2]. Moreover, this work also presents the ^13^C APT NMR and bi-dimensional NMR data, which is not available elsewhere for these substances. The combination of all these NMR results could clearly identify and distinguish the four cyclic PBT oligomers. 

#### 3.5.2. Assessment of Purity

^1^H-NMR was also used for the purity assessment of the isolated oligomers. A qNMR measurement method for the purity assessment of each isolated oligomer was applied using a certified reference material (CRM). DMSO_2_ has been selected as internal standard as it does not interfere with the resulting peaks from the studied PBT oligomers. 

The assessment of the purity of the isolated PBT oligomers (dimer up to pentamer) has been calculated according to the following equation [16,17,18,19]:

$$P_{\mathrm{Sample}}=\frac{I_{\mathrm{Analyte}}}{I_{\mathrm{CRM}}}\;.\;\frac{N_{\mathrm{CRM}}}{N_{\mathrm{Analyte}}}\;.\;\frac{M_{\mathrm{Analyte}}}{M_{\mathrm{CRM}}}\;.\;\frac{m_{\mathrm{CRM}}}{m_{\mathrm{CRM}}}\;.\;P_{\mathrm{CRM}}$$

where P_Sample_ is the purity of the sample as mass fraction, *P*_CRM_ the purity of the CRM as mass fraction, *I*_Analyte_ the integral of the analyte signal, *I*_CRM_ Integral of the CRM signal, *N*_Analyte_ the number of analyte protons, *N*_CRM_ the number of CRM protons, *M*_Analyte_ the molecular mass of the analyte, *M*_CRM_ the molecular mass of the CRM, *m*_Sample_ the mass of sample and *m*_CRM_ the mass of CRM.

Purities ranged from 96.1 (±1.2%) for the PBT tetramer to 97.0% (±1.1%) for the PBT trimer. Results of the purity assessment can be found in Supplementary Materials Table S1 and an example for the dimer spectrum with the presence of the DMSO_2_ is shown in Figure S4 in the Supplementary Material. All combined expanded uncertainties were calculated according to the Eurachem/CITAC Guide and reported as combined expanded uncertainties. Each of the uncertainty component is included in a cause-effect diagram (Figure S5) and was calculated separately for each isolated PBT oligomer (Table S1), using in all cases a coverage factor of 2 (k = 2) [16,17,18]. 

## 4. Conclusions

The lack of analytical standards with proper characterization and purity assessment is a considerable hindrance to the development and validation of an analytical method. This problem is even more serious when those substances are regulated in the EU, as it is the for PBT cyclic oligomers.

The method presented in this work proved to be efficient in producing pure oligomer standards. The purity of each oligomer was determined using a qNMR method with a certified reference material as internal standard. All the oligomers presented a very high purity ranging from 96.1% to 97% with expanded uncertainties spanning from 1.1 to 1.3%. Previously lacking physico-chemical information for this group of substances has also been provided, like solubility and the melting temperature for the cyclic pentamer.

## Figures and Tables

**Table 1:** Results of solubility studies for all four isolated PBT cyclic oligomers, from dimer to pentamer, according to WHO/FAO [10].

Solvent	PBT Cyclic Dimer	PBT Cyclic Trimer	PBT Cyclic Tetramer	PBT Cyclic Pentamer
MeOH	Insoluble	Insoluble	Insoluble	Insoluble
EtOH	Insoluble	Insoluble	Insoluble	Insoluble
IsOH	Insoluble	Insoluble	Insoluble	Insoluble
MeCN	Insoluble	Insoluble	Slightly soluble	Slightly soluble
DMF	Slightly soluble	Slightly soluble	Soluble	Soluble
DMA	Slightly soluble	Slightly soluble	Soluble	Soluble
DMSO	Slightly soluble	Very slightly soluble	Slightly soluble	Slightly soluble
HFIP	Freely soluble	Freely soluble	Freely soluble	Freely soluble
TFE	Soluble	Soluble	Freely soluble	Freely soluble
CH_2_Cl_2_	Very slightly soluble	Very slightly soluble	Soluble	Soluble
CHCl_3_	Soluble	Soluble	Soluble	Soluble

Note 1: solubility test procedures and solubility ranges adapted from WHO/FAO protocol [10].

**Table 2:** Differential scanning calorimetry (DSC) results for all four isolated PBT cyclic oligomers, from dimer to pentamer.

PBT	*DSC T*_m_ (°C)		Melting Point Apparatus (°C)
	(a)	(b)	
**Polymer**	224.1	226	223–225
**Cyclic dimer**	195.6	192.8	195–197
**Cyclic trimer**	167.5	166.7	166–168
**Cyclic tetramer**	246.7	246.8	246–248
**Cyclic pentamer**	215.6	NR	215–217

(a) values from the present study. (b) values reported by Wu et al. [15]. NR: values not reported.

**Table 3:** Chemical shifts in ppm for ^1^H and ^13^C NMR spectra of four PBT oligomers according to signal attribution of molecule.

PBT	^1^H			^13^C				
	a	b	c	e	d	a	b	c
**Cyclic dimer**	7.93	4.53	2.09	167.8	133.6	129.9	66.2	25.5
**Cyclic trimer**	8.12	4.52	2.04	168.0	133.8	130.1	66.2	25.0
**Cyclic tetramer**	8.11	4.52	2.03	168.0	133.6	129.9	66.4	25.1
**Cyclic pentamer**	8.13	4.52	2.02	168.0	133.6	129.9	66.3	25.1

(a) signal of terephthalate protons. (b,c) signal of methylenes of the oxybutylene chain. (d,e) signal of non-protonated carbons.

## Supplementary Materials

The following are available online at https://www.mdpi.com/2073-4360/11/3/464/s1. Figure S1. ^1^H-NMR spectrum of FCM 885 and 4 PBT cyclic dimer, trimer, tetramer & pentamer; (a) Mixture, (b) PBT dimer, (c) PBT trimer, (d) PBT tetramer, (e) PBT pentamer. Figure S2. 13C-NMR spectrum of FCM 885 and 4 PBT cyclic dimer, trimer, tetramer & pentamer; (a) Mixture, (b) PBT dimer, (c) PBT trimer, (d) PBT tetramer, (e) PBT pentamer. Figure S3. Monodimensional ^13^C-NMR and ^1^H-NMR of PBT cyclic dimer. Figure S4. qNMR spectra of the isolated PBT cyclic dimer, using dimethyl sulfone as internal standard. Figure S5. Cause-effect diagram of uncertainty contributions. Table S1. Purity and expanded uncertainty assessment for the isolated PBT oligomers by qNMR.
