# Supplementary material for: Isolation, Characterization and Structural Elucidation of Polybutylene Terephthalate Cyclic Oligomers and Purity Assessment Using a 1H qNMR Method
Source: Polymers (Basel). 2019 Mar 11;11(3):464. doi: 10.3390/polym11030464 (PMC6473883; doi:10.3390/polym11030464)
Supplement: Supplementary file 1 [file polymers-11-00464-s001.pdf]

Isolation, characterisation and structural elucidation of polybutylene terephthalate cyclic oligomers and purity assessment by a ^{1}H qNMR method

Emmanouil D. Tsochatzis, Joao Alberto Lopes*, Margaret V. Holland, Fabiano Reniero, Hendrik Emons, Claude Guillou

European Commission, Joint Research Centre (JRC), Directorate F, Via Enrico Fermi 2749, Ispra (VA), I-20127, Italy.

*Corresponding author: JRC-EURL-FCM@ec.europa.eu, Tel. +39 03322789782

Supplementary information

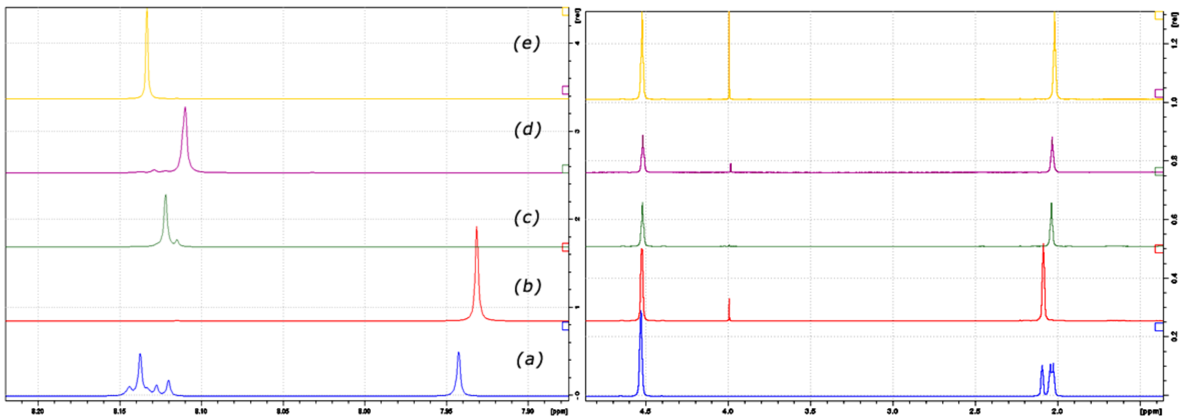

Figure S1. ^{1}H NMR spectrum of **FCM 885** and **4 PBT cyclic** dimer, trimer, tetramer and pentamer; (a) Mixture, (b) PBT dimer, (c) PBT trimer, (d) PBT tetramer, (e) PBT pentamer.

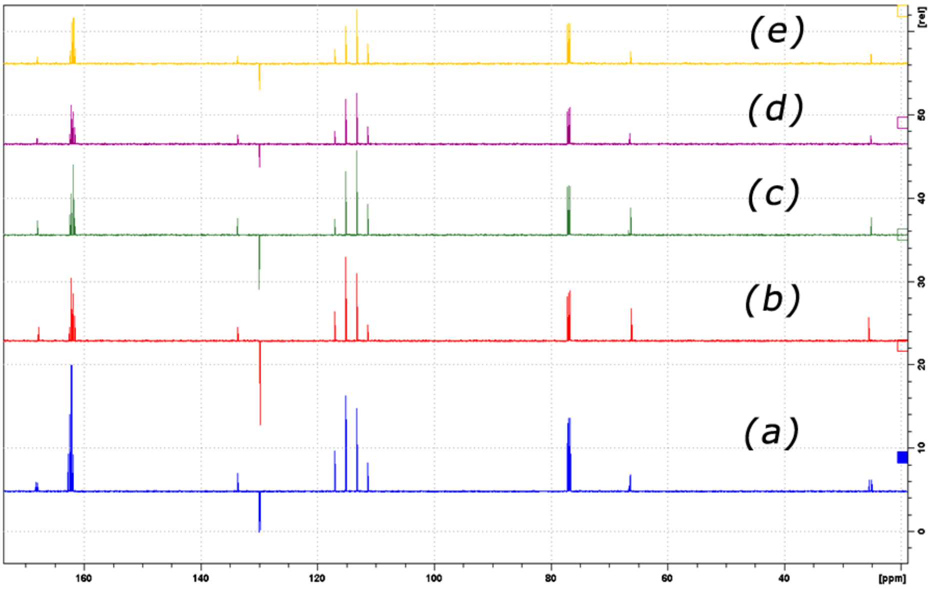

Figure S2. ^{13}C NMR spectrum of **FCM 885** and **4 PBT cyclic** dimer, trimer, tetramer and pentamer; (a) Mixture, (b) PBT dimer, (c) PBT trimer, (d) PBT tetramer, (e) PBT pentamer.

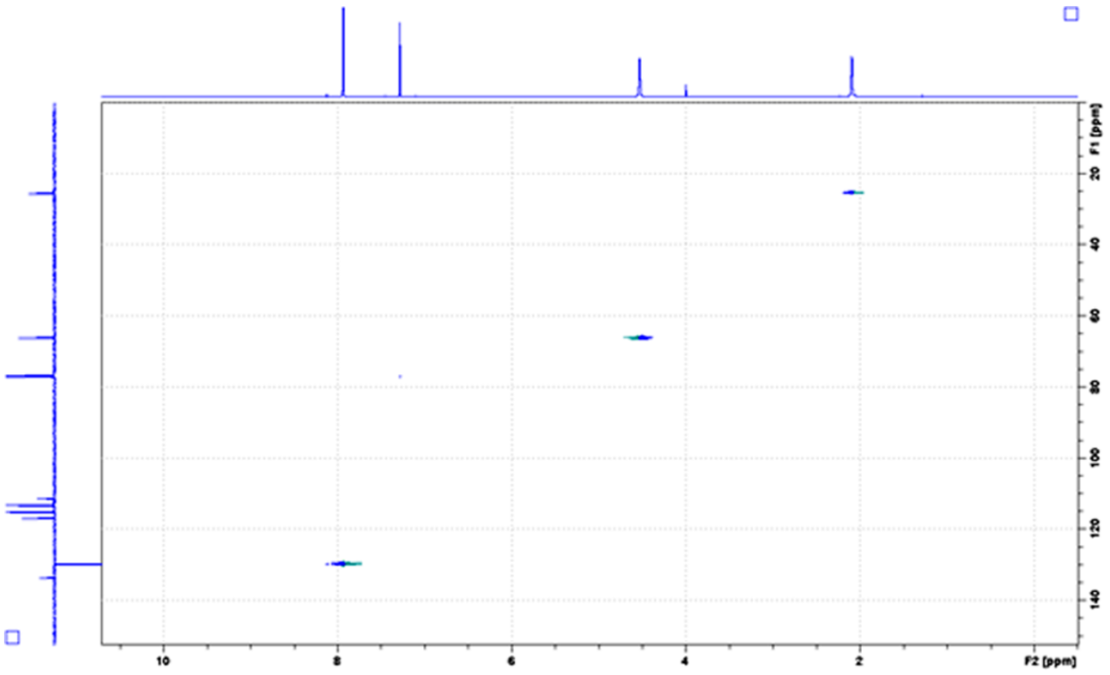

Figure S3: Monodimensional ^{13}C-NMR and ^{1}H-NMR of PBT cyclic dimer

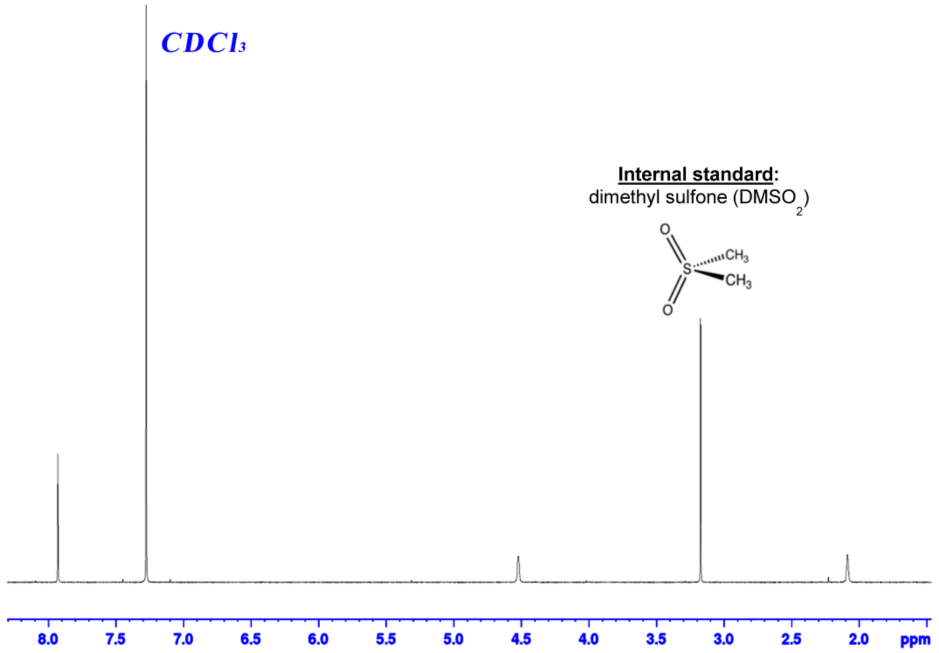

Figure S4. qNMR spectra of the PBT cyclic dimer, using dimethyl sulfone as internal standard.

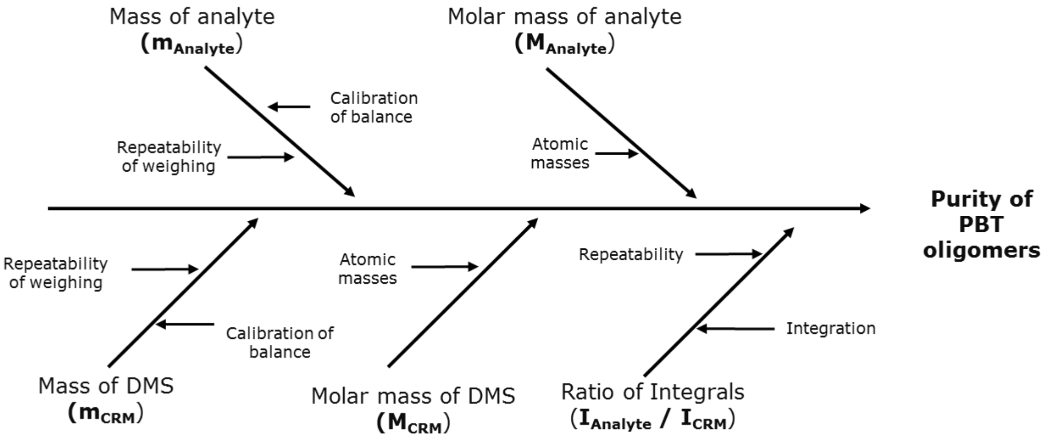

Figure S5. Cause-effect diagram of uncertainty contributions.

Table S1. Purity and expanded uncertainty assessment for the isolated PBT cyclic oligomers by qNMR.

	**PBT Cyclic dimer**		**PBT Cyclic trimer**		**PBT Cyclic tetramer**		**PBT Cyclic pentamer**	
	**Value**	**u (%)**	**Value**	**u (%)**	**Value**	**u (%)**	**Value**	**u (%)**
$I_{Analyte}$/I	9.9 (±0.05)	0.5 %	9.9 (±0.04)	0.4 %	10.4 (±0.05)	0.5	10.0 (±0.04)	0.4
I_{CRM}^{*}	18.3 (±0.08)	0.4 %	14.2 (±0.06)	0.4 %	14.6 (±0.05)	0.3	10.8 (±0.05)	0.5
N_{CRM}	6	0	6	0	6	0	6	0
$N_{Analyte}$	4	0	4	0	4	0	4	0
m_{CRM} (mg)	1.0	0.1	1.1	0.1	1.1	0.1	1.1	0.042 %
$m_{ANALYTE}$ (mg)	2.0	0.1	2.7	0.1	2.7	0.1	3.6	0.042 %
$M_{Analyte}$ (g/mol)	440.441	0.015 %	660.662	0.013 %	880.882	0.012 %	1101.103	0.011 %
M_{CRM} (g/mol)	94.13	0.001 %	94.13	0.001 %	94.13	0.001 %	94.13	0.001 %
P_{CRM} (DMS)%	99.96	0.15 %	99.96	0.15 %	99.96	0.15 %	99.96	0.15 %
**Purity (%)**	96.5		97.0		96.1		96.4	
**Relative Uncertainty (k=2); U (%)**	1.3 %		1.1 %		1.2 %		1.3 %	
